# Antitumor mechanisms and future clinical applications of the natural product triptolide

**DOI:** 10.1186/s12935-024-03336-y

**Published:** 2024-04-27

**Authors:** Shiwei Bao, Mei Yi, Bo Xiang, Pan Chen

**Affiliations:** 1 NHC Key Laboratory of Carcinogenesis, Hunan Provincial Cancer Hospital and the Affiliated Cancer Hospital of Xiangya School of Medicine, Central South University, Changsha, 410013 Hunan China; 2 The Key Laboratory of Carcinogenesis and Cancer Invasion of the Chinese Ministry of Education, Cancer Research Institute, School of Basic Medical Sciences, Central South University, Changsha, 410078 Hunan China; 3 FuRong Laboratory, Changsha, 410078 Hunan China; 4 Hunan Key Laboratory of Nonresolving Inflammation and Cancer, The Third Xiangya Hospital, Central South University, Changsha, 410013 Hunan China; 5 Department of Dermatology, Xiangya Hospital, Central South University, Changsha, 410008 Hunan China

**Keywords:** Natural product, Triptolide, Tumor, Molecular mechanism, Clinical application

## Abstract

Triptolide (TPL) is a compound sourced from Tripterygium wilfordii Hook. F., a traditional Chinese medicinal herb recognized for its impressive anti-inflammatory, anti-angiogenic, immunosuppressive, and antitumor qualities. Notwithstanding its favorable attributes, the precise mechanism through which TPL influences tumor cells remains enigmatic. Its toxicity and limited water solubility significantly impede the clinical application of TPL. We offer a comprehensive overview of recent research endeavors aimed at unraveling the antitumor mechanism of TPL in this review. Additionally, we briefly discuss current strategies to effectively manage the challenges associated with TPL in future clinical applications. By compiling this information, we aim to enhance the understanding of the underlying mechanisms involved in TPL and identify potential avenues for further advancement in antitumor therapy.

## Introduction

Traditional Chinese medicine boasts a rich history. Researchers worldwide are now actively studying and using numerous herbs in clinical treatments. Many FDA-approved drugs have drawn inspiration from compounds originating from Chinese herbs and minerals [1–3]. TPL, a diterpenoid tricyclic oxide derived from the herb Tripterygium wilfordii Hook F. (TwHF), stands as a prime example of such a compound. In traditional medicine, TwHF has been used to treat autoimmune and inflammatory diseases [4]. TPL demonstrates anti-inflammatory, anti-angiogenic, immunosuppressive, and antitumor properties [5, 6]. Additionally, it showcases neurotrophic and neuroprotective effects. Despite its promising clinical potential, TPL’s usage is impeded by its observed toxicity and lack of water solubility [4, 7].

Cancer has become a primary global concern, with incidence rates rising [8, 9]. Direct treatment options for cancer encompass a range of approaches, including surgery, chemotherapy, radiotherapy, immunotherapy, combination therapy, and targeted therapy [10]. Nonetheless, many advanced cancer patients still succumb to the illness. Only a tiny proportion of those in the early stages experience positive results, mainly because of the elevated rate of recurrence [11]. On the whole, the survival rates among cancer patients continue to be low due to the absence of effective treatments. Discovering a cure for human cancer remains a substantial challenge. TPL, emerging as a promising candidate in tumor therapy research, has been recognized in numerous studies for its potent antitumor capabilities against various malignancies, including lung, pancreatic, nasopharyngeal, breast, stomach, liver, and colon cancers [12]. In clinical application research, TPL-related studies in various applied forms have been carried out in the laboratory, and two drugs are currently in clinical trials [13]. This review aims to briefly present the molecular mechanisms that drive TPL’s antitumor effects and its possible clinical applications. Our comprehensive analysis will deepen our comprehension of TPL’s antitumor impacts and its potential in cancer treatment.

## Anti-inflammatory effects of TPL

Inflammation plays a significant role in the progression of tumors. It represents immune response to injury, infection, and tissue damage, involving complex interactions among cells and molecules. These interactions aid in tissue repair and offer protection against infection by involving the infiltration and activation of immune cells [14]. However, persistent or chronic inflammation can negatively influence tumor development. Research has demonstrated that persistent inflammation can induce apoptosis and abnormal cell proliferation, as well as facilitate the invasion and angiogenesis of tumor cells [15, 16].

TPL exerts inhibitory effects on the release of plasma inflammatory cytokines like TNF-α [17], IL-1β, IL-6 [18], MCP-1 [19], MMP-3, MMP-9 [20], Cox-2, NLRP3 [21], and others. In the context of bone-associated inflammation, TPL demonstrates significant anti-inflammatory properties [18]. Following TPL treatment, a noticeable reduction in F4/80^+^ macrophages and CD3^+^ T cells was observed within inflammatory lesions [22]. After TPL treatment, malondialdehyde and reactive oxygen species (ROS) were noticeably increased, as were superoxide dismutase and glutathione (GSH) activities. It effectively curtails oxidative stress. In the case of collagen-induced arthritis (CIA), the antioxidant effect of TPL has potential therapeutic value [23]. Huang et al. found that TPL addresses the Treg/Th17 imbalance in CIA mice [24]. TPL is implicated in inhibiting the activation of the JNK/PTEN-STAT3 signaling pathway, which attenuates bone damage and reduces the inflammatory response within the bone in mice. Furthermore, the encapsulation of TPL with targeted nanoparticles enhances its anti-inflammatory properties and reduces toxicity [22]. Rheumatoid arthritis (RA) is a persistent autoimmune ailment with fibroblast-like synoviocyte (FLS) proliferation and inflammatory infiltration [25]. In RA-fibroblast-like synovial (RA-FLS) cells, TPL was observed to decrease TNF-α-induced phosphorylated JNK expression, block the JNK MAPK pathway, and inhibit RA-FLS cell migration and invasion [26]. Long non-coding RNA (lncRNA) has also been shown to have a relevant role in the progression of inflammation. Research has shown that TPL can modulate lncRNA ENST00000619282 to diminish inflammatory infiltration in FLS cells [27]. The expression of ENST00000619282 was notably elevated in peripheral blood mononuclear cells (PBMCs) obtained from RA patients. The overexpression of ENST00000619282 led to increased levels of caspase3, caspase8, Fas, FasL, and Bax, while decreasing the levels of Bax-x1 and Bcl-2. TPL was able to lower down-regulate ENST00000619282, leading to the promotion of apoptosis and attenuation of the inflammatory response associated with RA [27]. This may be the mechanism by which TPL exerts anti-RA effects. Significantly, in rats undergoing deep hypothermia circulatory arrest (DHCA), TPL exhibits neuroprotective effects by activating the NRF2/NQO-1/HO-1 pathway and suppressing the activity of NF-κB p65, thereby acting as an anti-inflammatory agent [18]. Tang et al. also observed that TPL induces the activation of the NRF2/HO-1 signaling pathway while concurrently inhibiting the PDE4B/AKT/NF-kB pathway [28]. This dual mechanism leads to reduced ROS production, restrained macrophage infiltration, mitigated M1-type polarization, and alleviated intestinal inflammatory responses. Additionally, TPL inhibits the expression of the chemokines CCL2 and CCR2, which promotes M2-type polarization of macrophages and reduces intracellular inflammatory factor levels [29]. Unlike other phagocytes, neutrophils can form neutrophil extracellular traps (NETs), releasing various inflammatory substances associated with inflammatory diseases [30–32]. The researchers isolated peripheral blood neutrophils from volunteers, subjected the cells to TPL treatment, and assessed the levels of NETs. TPL has been found to inhibit NET formation, which may be a manifestation of its anti-inflammatory effect [33]. Moreover, pretreatment of dendritic cells (DCs) with TPL induces their transformation into tolerant DCs [34]. Indeed, this led to the development of a number of responses, such as a decrease in the number of CD4^+^ T cells in the spleen and mesenteric lymph nodes, an increase in the number of Treg cells, a remodeling of the immune microenvironment, and a reduction in the inflammatory response in mice with colitis.

There have been no clear reports suggesting that the antitumor effect of TPL is directly related to the anti-inflammatory effect. However, based on the important role of inflammation in the development of tumors, the concomitant function of TPL against inflammation (Table 1) could indirectly play an antitumor role.

**Table 1:** Anti-inflammatory effects of TPL

Target	Mode of action	References
Inflammatory Cytokines	Inhibits release	[17–21]
Macrophages/T cells	Reduces infiltration	[22]
Oxidative Stress Molecules	Alleviates oxidative stress	[23]
Treg/Th17 Ratio	Corrects imbalance	[24]
JNK/STAT3 Pathway	Inhibits activation	[22]
JNK MAPK Pathway	Blocks pathway	[16]
lncRNA	Downregulates expression	[27]
NRF2/NF-κB Pathways	Modulates pathways	[18]
PDE4B/AKT/NF-kB pathway	Inhibits pathway	[28]
CCL2/CCR2	Inhibits expression	[29]
NETx	Inhibits formation	[30–33]
Dendritic Cells	Induces tolerogenic transformation	[34]

## Anti-angiogenic effect of TPL

Network pharmacology studies have demonstrated that TPL can influence angiogenesis through signaling networks [35]. Pathological angiogenesis is a hallmark of cancer and can occur at any stage of the disease [36]. Tumor angiogenic activity is closely linked to prognosis, and the development of anti-angiogenic drugs could not only be used for cancer treatment but also aid in preventing tumor recurrence and metastasis [37]. Elucidating the molecular mechanisms underlying TPL’s anti-angiogenic effects would facilitate the development and application of related drugs.

The study by Kong et al. using a CIA model in rats demonstrated that TPL could inhibit the expression of pro-angiogenic factors including TNF-α, IL-17, VEGF, VEGFR, Ang-1, Ang-2, and Tie2. It also suppressed the activation of the downstream mitogen-activated protein kinase signaling pathway, thereby exerting an anti-angiogenic effect in RA [38]. This may also be the mechanism by which TPL exerts its anti-angiogenic effects in tumors.

In a study on hepatocellular carcinoma, the authors found that TPL significantly inhibited the in vitro angiogenic ability of HepG2 cells [39]. This effect was accompanied by the downregulation of serine palmitoyltransferase long chain base subunit 2 (SPTLC2) expression and a reduction in sphingosine-1-phosphate (S1P) production, which may be one of the key mechanisms underlying TPL’s anti-angiogenic action. Furthermore, TPL inhibited the activation of the NF-κB signaling pathway, directly suppressing the expression of VEGF in HUVECs, breast cancer cells MDAMB-231 and MCF-7, thereby affecting tumor cell angiogenesis [40–42]. In a laser-induced choroidal neovascularization (CNV) murine model, TPL inhibited the M2 polarization of macrophages and reduced the expression of angiogenic and inflammatory factors such as VEGF [41]. In a breast cancer mouse model, the combination of TPL and cisplatin (DDP) significantly inhibited the production of VEGF-related protein clusters of differentiation 31 (CD31) and CD105 [43]. This combination therapy exhibited better efficacy than TPL alone. The inhibitory effect of TPL on VEGF expression was also validated in osteosarcoma, which may be related to the suppression of the Wnt/β-catenin signaling pathway and the subsequent induction of autophagy in tumor cells [44]. The attenuated Salmonella strain VNP20009 as a monotherapy has shown promising efficacy in melanoma. However, its clinical application is often hampered by the reduced tumor-colonizing ability of VNP20009, which significantly diminishes its antitumor potency. Co-treatment with TPL can reduce the infiltration of neutrophils in melanoma and significantly improve the tumor colonization of VNP20009 [45]. Simultaneously, the combination synergistically downregulates the expression of VEGF, inhibiting angiogenesis. Compared to VNP20009 monotherapy, the combination treatment exhibited significantly enhanced efficacy.

These findings may provide an explanation for the anti-angiogenic effects of TPL in cancer treatment. In the study by He et al., TPL exhibited anti-angiogenic activity with an IC50 of 45 nM in vitro. In vivo, a concentration of 100 nM showed a highly significant effect, and the optimal inhibitory concentration of TPL was 0.75 mg/kg/day in a mouse tumor xenograft model [46].

## Antitumor effect of TPL

### TPL-related classical signaling pathways

**Fig. 1:**
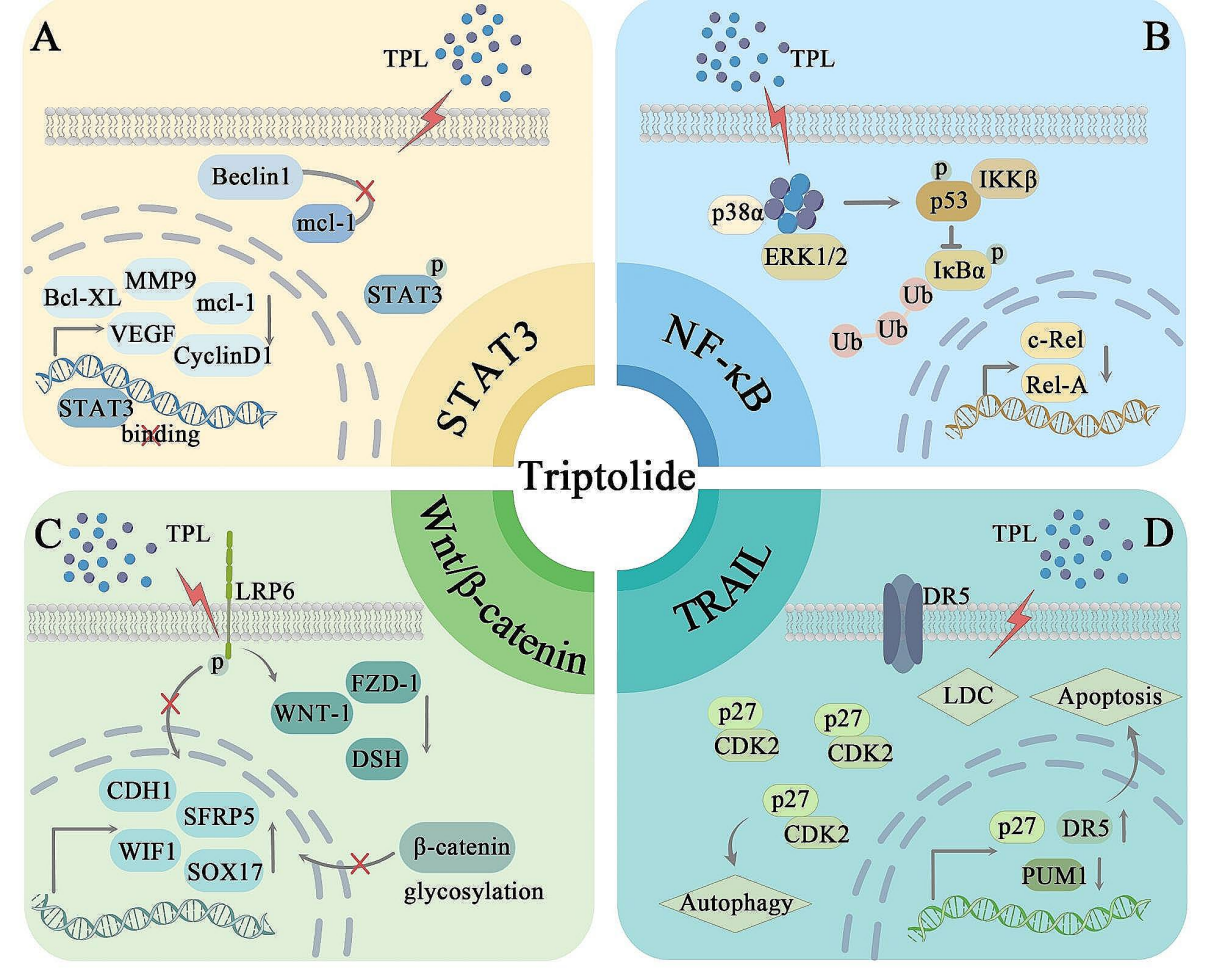
Main signaling pathways involved in TPL in cancer. (**A**)TPL dampens the activation of the STAT3 signaling pathway by preventing STAT3 from binding to DNA. Simultaneously, it disrupts the binding interaction between Beclin1 and Mcl-1, resulting in decreased expression of genes controlled by STAT3, which are associated with anti-apoptotic, proliferative, and angiogenic functions. (**B**)TPL effectively inhibits NF-κB activity and reduces the protein expression of its subunits, c-Rel and Rel-A. Simultaneously, TPL binds to and activates p38α and ERK1/2, stabilizes p53, and inhibits IκBα phosphorylation. (**C**)TPL hinders the Wnt/β-catenin signaling pathway through the suppression of LRP6 phosphorylation, subsequently inhibiting DSH activation. This action leads to heightened expression of CDH1, WIF1, and other related factors. Additionally, TPL induces demethylation to provide further inhibition of the Wnt signaling pathway. (**D**)TPL enhances TRAIL-related signaling activation by increasing DR5 expression and reducing PUM1 expression, rendering cells more sensitive to apoptosis. It leads to an increase in p27 and CDK2 complexes, inducing autophagy. When combined with TRAIL, TPL causes lysosome-dependent cell death

TPL is involved in several classical signaling pathways during tumorigenesis and development, and Fig. 1 illustrates several main signaling pathways involving TPL.

NF-κB is as a pivotal regulator in numerous processes governing gene expression, and also in cancer cell proliferation, migration and apoptosis [47]. Aberrant activation of NF-κB has been documented in a multitude of human malignancies [47, 48]. TPL has been recognized as a potent inhibitor of NF-κB activation [49, 50]. In experimental studies on pancreatic cancer transplant tumors, hypoxia is a marker for aggressive growth and spontaneous metastasis formation [51, 52]. TPL treatment efficiently reduces the NF-κB binding activity induced by hypoxia. This, in turn, inhibits the expression of its subunits, specifically c-Rel and Rel-A proteins [53]. TPL binds to and triggers the activation of p38α and extracellular regulated protein kinases 1/2 (ERK1/2) [54, 55]. It leads to the phosphorylation and stabilization of p53. In turn, p53 competes with IκBα, a suppressor protein of NF-κB, for binding to IKKβ. This process hinders the phosphorylation and breakdown of IκBα, effectively preventing the nuclear translocation of NF-κB. The use of TPL disrupts the H19/MiR-204-5p/NF-κB/FLIP axis, resulting in the non-proteasome-mediated degradation of FLIPS and heightened apoptosis in TNF-α-stimulated tumor cells [56, 57]. When combined with this axis disruption, TPL significantly augments its anticancer impact.

Signal transducer and activator of transcription 3 (STAT3) is strongly linked to both inflammation and tumorigenesis [58, 59]. Kim et al. explored the impact of TPL on STAT3 [60, 61]. They discovered that it suppressed STAT3 activation, diminished STAT3 phosphorylation, hindered STAT3 DNA binding, and lowered the expression of genes regulated by STAT3, including antiapoptotic genes (Bcl-xL and mcl-1), proliferative genes (CyclinD1), matrix metallopeptidase 9 (MMP-9), and angiogenic genes (VEGF). In cisplatin-resistant SKOV3/DDP tumor cells, Zhong et al. showcased how TPL restrained the JAK2/STAT3 signaling pathway [62]. Furthermore, it interfered with the connection between Mcl-1 and Beclin1, promoting a type of autophagy lethal to cancer cells. TPL mitigates the inflammatory response by inhibiting the mTOR/STAT3 signaling pathway. It encourages a shift from M1 to M2 polarization and reduces the activation of RAW 264.7 macrophages induced by lipopolysaccharide (LPS) [63].

In prostate cancer, Hu et al. revealed that TPL treatment increased the expression of death receptor 5 (DR5), rendering cancer cells significantly sensitive to apoptosis mediated by TRAIL [64]. TPL treatment downregulated PUM1 expression and increased apoptosis. Additionally, it upregulated p27 expression, increasing p27-CDK2 complexes and enhancing TRAIL-induced cellular autophagy for antitumor purposes [65]. In pancreatic cancer cells, the coadministration of TRAIL and TPL elicited heightened lysosomal membrane permeability and prompted lysosome-dependent cell death (LCD) [66].

Revitalization of the Wnt/β-catenin pathway is intricately linked to tumor progression [67, 68]. This classical signaling pathway involves the transcription factor β-catenin, which mediates gene activation [69]. The overexpression of Wnt pathway molecules has been correlated with an unfavorable prognosis in non-small cell lung cancer (NSCLC), as evidenced in a study conducted by Reno et al. [70, 71]. TPL treatment has been shown to decrease the methylation level of the WIF1 promoter and increase its expression, leading to the suppression of Wnt pathway activity [72]. Within the pancreatic cancer cell lines MIAPaca-2 and S2-VP10, TPL hampers the phosphorylation of the Wnt signaling receptor LRP6 [73]. This, in turn, disrupts the activation of WNT_1, FZD_1, and DSH. Additionally, TPL inhibits β-catenin glycosylation and prevents the nuclear translocation of β-catenin. TPL increased the expression of multiple Wnt repressor proteins, causing histone 3 global epigenetic changes that induced apoptosis in lung cancer cells. Moreover, TPL had no impact on the DNA methylation status of CpG islands within the promoter region of the Wnt repressor [74, 75]. Notably, this suppression of the Wnt signaling pathway intensifies with higher concentrations of TPL. In T-cell acute lymphoblastic leukemia (T-ALL), TPL stimulates the expression of Wnt pathway inhibitors, including WIF1, SOX17, CDH1, and SFRP5 [76]. Additionally, it facilitates the demethylation of these genes to prevent abnormal epigenetic alterations within the Wnt signaling pathway, ultimately inhibiting the progression of T-ALL.

### TPL affects transcription and epigenetics

After TPL treatment, there is a decrease in the phosphorylation level of Ser2, resulting in the transcriptional inhibition of RNA polymerase II (RNAPII) and subsequent diminished transcriptional activity of RNA polymerase I (RNAPI) [77]. TPL targets XPB, a subunit of the universal transcription factor TFIIH that plays a role in RNAPII-mediated transcription initiation. TPL forms covalent adducts with Cys342 at the active site of XPB, inhibiting its DNA-dependent ATPase activity [78–82]. This inhibition leads to hyperphosphorylation of Rpb1, the largest subunit of RNAPII, and reduces Rpb1 levels in cancer cells. Consequently, TPL mediates the degradation of RNAPII, increases drug sensitivity, and induces cancer cell death. TPL treatment also inhibits the transcription of RNA polymerase III (RNAPIII) [83, 84]. In tumor cells, the expression of tRNAs and 5 S rRNA, which are products of RNAPIII, is increased. Liang et al. conducted a study on the impact of TPL in colorectal tumors [85]. They discovered that TPL disrupts the interactions between TBP and Brf1, consequently decreasing the assembly of TFIIIB on tRNA and 5 S rRNA promoters. This disruption leads to the suppression of RNAPIII transcription, ultimately resulting in the inhibition of tumor growth.

Super enhancer has extremely high transcriptional activation capacity, which is essential in the progression of tumor development and is currently a more studied direction of epigenetic regulation [86]. In pancreatic ductal adenocarcinoma (PDAC), TPL triggers the downregulation of super enhancer (SE)-related gene expression in both pancreatic tumor cells and cancer-associated fibroblasts (CAFs) [87]. This disruption of the super enhancer system leads to a reprogramming of cellular cross-talk and signaling within PDAC, ultimately inducing antitumor activity. Hence, TPL holds the potential to offer an efficient therapeutic option for individuals diagnosed with pancreatic cancer by instigating epigenetic reprogramming. In the realm of cancer development, enhancer of zeste homolog 2 (EZH2) serves as a crucial histone methyltransferase [88, 89]. Research has demonstrated that TPL hinders the growth of prostate cancer (PCa) cells and reduces the expression of EZH2 [90]. After subjecting PC-3 prostate cancer cells to TPL treatment, we observed an upregulation in the mRNA expression levels of specific target genes (ADRB2, DAB2IP and CDKN2A) that are under negative regulation by EZH2. Conversely, the mRNA levels of target genes (such as cyclin D1) that are positively modulated by EZH2 exhibit a decrease. Adenosquamous carcinoma of the pancreas (ASCP) represents an exceptionally aggressive variant of pancreatic cancer, propelled by the activation of super enhancers and characterized by elevated expression levels of the MYC gene [91]. Minnelide, a pharmaceutical product derived from TPL, is an oral medication designed to combat super enhancers and subsequently lower MYC expression [92]. It is presently undergoing clinical phase II trials.

### TPL and cell death

**Fig. 2:**
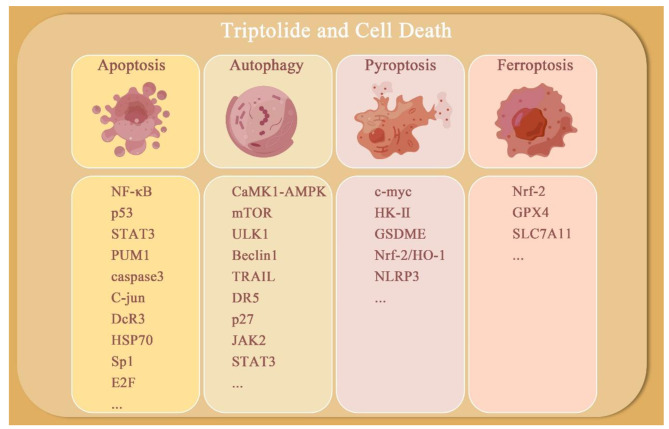
TPL induces cell death. TPL generates multiple modes of cell death, such as apoptosis, autophagy, pyroptosis and ferroptosis. The primary targets have been outlined in the figure

The connection between TPL and the death of tumor cells encompasses the initiation of various processes, including apoptosis, autophagy, pyroptosis, and ferroptosis as shown in Fig. 2. Delving deeper into the precise molecular mechanisms that underlie these outcomes will yield invaluable insights for crafting targeted anticancer treatments utilizing TPL. In this context and Table 2, we provide a succinct overview of the mechanisms through which TPL triggers cellular demise, as documented in existing published research.

**Table 2:** Targets of TPL in different cancers

Cell Death	Target	Cancer Types	References
Apoptosis	NF-κB, p53	Pancreatic Cancer, Gastric Cancer, Hepatoma, Lung Cancer	[51–57]
	STAT3	Multiple Myeloma, Non-small Cell Lung Cancer	[60, 61]
	PUM1	Pancreatic Cancer	[65]
	Akt/Hdn2	Prostate Cancer	[64]
	MDM2, p85	Breast Cancer	[93, 94]
	CaMKKβ/Ampk	Lung Cancer	[95, 96]
	P53, caspase3, caspase9	Gastric Cancer	[97–99]
	CDKN1A, C-jun, NF-κB, p65	Thyroid Cancer	[100]
	HSP70	Pancreatic Cancer	[101]
	Sp1, HSP70	Pancreatic Cancer	[102]
	miR-142-3p, HSP70	Pancreatic Cancer	[103]
	DcR3	Pancreatic Cancer	[104]
	DcR3, MTA1	Oral Cancer	[105]
	E2F	Colon Cancer	[85]
Autophagy	CaMK1-AMPK, mTOR, ULK1, Beclin1	Prostate Cancer, Neuroblastoma	[106–108]
	JAK2/STAT3	Ovarian Cancer	[62]
	TRAIL, DR5, p27	Prostate Cancer	[65, 66]
Pyroptosis	c-myc, HK-II, GSDME	Head and neck squamous cancer	[109]
Ferroptosis	Nrf-2, GPX4, SLC7A11	Leukemia	[110–112]

#### TPL causes apoptosis

In a study by Phillips et al., analysis of four apoptotic markers, including Annexin V, TUNEL, caspase-3 activity, and mitochondrial cytochrome c, was performed using two pancreatic cancer cell lines. The research findings suggest that TPL treatment led to a notable upsurge in Annexin V expression, intensified TUNEL staining, heightened caspase-3 activity, and the release of mitochondrial cytochrome c in both pancreatic cancer MiaPaCa-2 and PANC-1 cells [101]. These observations suggest that TPL can trigger apoptosis in pancreatic cancer cells. Moreover, this TPL-induced apoptotic effect has been documented in diverse tumor cell lines.

TPL treatment effectively suppresses the Akt/Hdn2 signaling pathway, rendering prostate cancer cells more susceptible to TRAIL-mediated apoptosis [64]. MDM2 is a repressor of p53. TPL inhibits the expression of MDM2, resulting in the transcription factor REST not properly binding to MDM2, a marked increase in the expression of the p85 regulatory subunit of PI3-kinase, and inhibition of Akt activation [93, 94]. When TPL is employed in conjunction with Nutlin-3a (an MDM2 inhibitor), it results in a reduction in the mRNA levels of XIAP and Mcl-1 in p53 wild-type cells. Simultaneously, it boosts the transcriptional expression of PUMA and p21, which are downstream targets of p53 [113]. This combined treatment inhibits the proliferation of p53 wild-type AML cells and induces apoptosis through mitochondria-mediated mechanisms.

TPL additionally modulates intracellular calcium ion (Ca^2+^) levels. It activates the Ca^2+^/CaMKKβ/AMPK signaling pathway, increasing AMPK phosphorylation and decreasing AKT phosphorylation, specifically at the S473 and T308 sites [95, 96]. These molecular changes ultimately culminate in apoptosis. In HCT-116 colorectal carcinoma cells, Liskova et al. observed that silencing the mitochondrial division-associated protein Drp1 led to a decreased apoptosis following treatment with TPL [114].

TPL demonstrates pronounced growth inhibition and triggers apoptosis in MKN-45 cells with wild-type p53. However, it does not exhibit the same effects in gastric cancer MKN-28 and SGC-7901 cells harboring mutant p53 [97]. This underscores the importance of functional p53 in mediating the antitumor effects of TPL. TPL induces the phosphorylation of the p53 protein and elevates its nuclear presence, resulting in heightened activation of apoptosis regulators, including caspase-9 and caspase-3 [98, 99]. Furthermore, TPL suppresses the expression of Bcl-2, consequently facilitating apoptosis. The use of p53 inhibitors can prevent these effects. The use of the pan-caspase inhibitor and caspase-3-specific inhibitor (zVAD-fmk, DEVD-fmk) has led to the inhibition of caspase-3 cleavage and a substantial reduction in TPL-induced apoptosis, as demonstrated in studies [97]. Wang et al. showed that TPL upregulates the protein expression of CDKN1A and phosphorylated p53 while reducing the levels of phosphorylated c-jun and phosphorylated NF-κB p65 [100]. These molecular changes result in inhibiting thyroid cancer cell growth and the induction of apoptosis. In an experimental study conducted by Dai et al., TPL treatment was found to impede DNA damage repair, promote apoptosis, and augment the radiosensitivity of radiation-induced lung cancer cells [115]. In Wang et al.‘s study, it was observed that TPL did not effectively activate the p53 signaling pathway [116]. However, it did upregulate caspase-3 and caspase-9, while concurrently downregulating bcl-2 expression. Interestingly, the level of bax remained unchanged under TPL treatment. Their research delved into the antitumor properties of TPL in human endometrial cancer cells, specifically HEC-1B cells, while also investigating the mechanisms that underlie these effects. Significantly, the utilization of z-VAD-fmk markedly attenuated the cytotoxic impact of TPL on HEC-1B cell proliferation in a dose-and-time-dependent manner. This implies that TPL has the capacity to impact the apoptosis of endometrial cells through a mitochondrial pathway that operates independently of p53. This potential characteristic of TPL as a chemotherapeutic agent is undoubtedly worth further exploration and consideration.

Heat shock protein 70 (HSP70) is a pivotal molecular chaperone that plays a critical role in facilitating proper protein folding and the refolding of misfolded proteins. In doing so, it contributes significantly to the maintenance of protein homeostasis, which has a direct and profound impact on human health [117, 118]. Elevated levels of HSP70 enable cells to withstand and survive even in the face of severe cellular injury [119]. Numerous studies have consistently demonstrated that HSP70 exhibits high expression levels in various malignancies, including breast, colon, liver, and cervical cancer, among others [120–125]. TPL has been found to significantly reduce HSP70 mRNA and protein expression levels in pancreatic cancer cells. The decrease in HSP70 expression is associated with the initiation of caspase-dependent apoptotic cell death, particularly in pancreatic cancer cells. Notably, this reduction in HSP70 protein expression does not affect normal pancreatic ductal cells [101]. The underlying mechanism has been thoroughly investigated by Banerjee et al. It has been observed that TPL impedes the glycosylation of the transcription factor Sp1, thus preventing its nuclear localization, impairing its DNA-binding capability, and consequently suppressing the expression of HSP70 [102]. MacKenzie et al. also discoverd that TPL induces an elevated expression of miR-142-3p, which functions as a negative regulator of HSP70 [103]. By inhibiting HSP70 expression, TPL triggers apoptosis in tumor cells.

Abnormal expression of decoy receptor 3 (DcR3) promotes tumor growth and represents one of the targets for cancer diagnosis and treatment [104, 126]. TPL, as a promising therapeutic candidate, hampers the expression of DcR3 and triggers apoptosis in pancreatic cancer cells. Targeting DcR3 expression heightens the susceptibility of pancreatic cancer cells to TPL-induced apoptosis [104]. Furthermore, in vivo studies utilizing DcR3 siRNA showcase a substantial augmentation of TPL-induced apoptosis and the inhibition of tumor growth. Yang et al. conducted research investigating the impact of TPL on oral cancer and noted that it exerts an inhibitory influence on the expression of DcR3 and the transcription factor metastasis-associated protein 1 (MTA1) in ex vivo and preclinical patient-derived xenograft tumor (PDTX) models [105]. This mechanism contributes to its anticancer effects in the context of oral cancer.

TPL treatment induces cell cycle arrest, allowing cells additional time for damage repair [127, 128]. In the RPMI8226 multiple myeloma cell line, TPL induces cell cycle arrest at the G0/G1 phase and prompts apoptosis [129]. In gallbladder carcinoma, TPL treatment induces an S-phase block [130]. In colon cancer HCT116 cells, TPL induces G2 phase blockage and apoptosis [85]. Research has shown that lower concentrations of TPL (10 nM) trigger cell aggregation in the G0/G1 phase, potentially regulating downstream DNA binding events. This results in the suppression of E2F-mediated transcriptional activation and brings about cell cycle arrest specifically in the G1 phase.

Conversely, when higher concentrations of TPL (> 20 nM) are applied, they lead to an expanded population of cells in the sub-G1 phase, a hallmark of apoptotic cells [85, 131]. TPL has a dose-dependent suppressive effect on the cell cycle [132]. TPL promotes the expression of P21 (cell cycle repressor) and Bax (pro-apoptotic factor), which promotes the development of apoptosis in glioblastomas. These effects are likely associated with the inhibition of PROX1 transcription by TPL.

#### TPL causes autophagy

Autophagy is a crucial process in maintaining the normal metabolism of tissues and organs, and any dysfunction or impairment in autophagy can result in various pathological alterations [133, 134]. Additionally, it can make individuals more susceptible to the development of cancer cells [135]. Research findings have indicated that TPL exerts its anticancer effects on various types of pancreatic cancer cells through two distinct pathways. In certain cell lines, it triggers caspase-dependent apoptotic cell death, while in others, it initiates caspase-independent autophagic pathways. Within pancreatic cancer cells, there is a dynamic interplay between these two cell death pathways. These pathways can function independently, with autophagy being the primary mechanism of cell death. Alternatively, autophagy can counteract apoptosis, with apoptosis becoming noticeable only when autophagy is inhibited or suppressed. The mechanism of TPL’s action has been explored in both neuroblastoma and prostate cancer [106, 107]. These studies have demonstrated that TPL treatment results in elevated levels of the light chain 3II (LC3II) protein within cells. Consequently, this triggers the activation of the CaMK1-AMPK signaling pathway. This signaling cascade leads to the inhibition of mTOR, activation of ULK1, and upregulation of Beclin1, ultimately inducing autophagy. In cutaneous squamous cell carcinoma (cSCC), TPL inhibits the Akt/mTOR signaling pathway, promoting apoptosis and autophagy [108].

#### TPL causes pyroptosis

Cai et al. conducted research to elucidate the mechanism of action of TPL in head and neck cancer (HNSC) [109]. They discovered that TPL induces the death of HNSC cells through GSDME-induced cellular pyroptosis. Moreover, TPL’s impact on HNSC cells involves the inhibition of the c-myc/HK-II axis, which results in a decrease in HK-II on mitochondria. This, in turn, activates the Bad/Bax-Caspase 3 cascade, ultimately leading to cellular death. This finding was the first to demonstrate that TPL can induce pyroptosis. In another study investigating the mechanism of TPL in liver injury, Han et al. found that TPL could trigger pyroptosis in Kupffer cells [136]. TPL binds to the Caspase-3-VAL27 site, leading to the cleavage of Caspase-3. Cleaved-Caspase-3 promotes GSDME cleavage, thereby inducing pyroptosis in Kupffer cells. Additionally, Lv et al. conducted a study on diabetic nephropathy (DN), where TPL was found to inhibit pyroptosis [137]. TPL reduces oxidative stress (OS) and ROS through activation of the Nrf-2/HO-1 pathway and attenuates cellular pyroptosis by inhibiting the NLRP3 inflammatory vesicle pathway [137]. However, the association between TPL and pyroptosis in different diseases requires further investigation.

#### TPL causes ferroptosis

In a study investigating leukemia resistance to the chemotherapeutic drug doxorubicin (Dox), researchers established Dox-resistant cell lines, both K562 and HL-60, by exposing them to low doses of Dox [110]. They observed a significant upregulation of erythroid 2-related factor 2 (Nrf2) expression in these Dox-resistant cell lines, as well as in clinical specimens. Nrf2 is essential in the antioxidant response. Silencing Nrf2 increases the sensitivity of leukemia cells to Dox. Furthermore, treatment with TPL effectively inhibited Nrf2 expression, resulting in elevated levels of reactive oxygen species (ROS), reduced lipid oxidation, and decreased expression of GSH peroxidase 4 (GPX4). Additionally, TPL induced iron-mediated cell death in leukemia cells and reinstated sensitivity to Dox-resistant leukemia cells. Fang et al. devised a nanoplatform that relies on both acid and GSH sensitivity to augment cancer therapy by harnessing the synergistic effects of “1 + 1” apoptosis and “1 + 1” ferroptosis [111]. In this system, ZIF8 serves a dual purpose: it improves drug targeting and prevents premature drug degradation. Within the center of PtIV, a significant quantity of GSH undergoes reduction to form cisplatin, leading to the release of heme and TPL. This process effectively inhibits GPX4 activation. Indeed, in this innovative approach, cisplatin and heme work in tandem to induce “1 + 1” apoptosis through a combination of chemotherapy and photodynamic therapy. Concurrently, TPL plays a crucial role by modulating Nrf2, leading to the inhibition of GSH expression. This, in turn, amplifies membrane lipid peroxidation, ultimately culminating in the achievement of “1 + 1” ferroptosis. This dual mechanism effectively targets cancer cells, enhancing the therapeutic potential of the treatment. This experiment was validated in a breast cancer model. Moreover, TPL directly binds to SLC7A11, leading to the inactivation of the SLC7A11/GPX4 signaling pathway, thus triggering ferroptosis and causing TPL-induced cardiotoxicity [112]. This study elucidates the relationship between TPL and ferroptosis.

### Alternative pathways of action of TPL

The administration of TPL often results in altered nuclear morphology in tumor cells. Leuenroth et al. treated Hela cells with 100 nmol/L TPL and observed a significant compromise in nucleolus integrity, leading to potent transcriptional repression and subsequent cell death [77]. This damage was reversible within 4 h of TPL incubation but eventually led to direct cell death after 6 h. Additionally, nuclear spots became notably rounded and enlarged after 2 h of TPL incubation, serving as a morphological indication of transcriptional arrest. These findings suggest an alternative mechanism by which TPL exerts its antitumor effects.

Xie et al. showed that TPL notably downregulated the expression of programmed death ligand 1 (PD-L1) in NSCLC cells [138]. This modulation of PD-L1 expression could have implications for immunotherapy approaches in NSCLC treatment. TPL treatment inhibited the EGFR signaling pathway in NSCLC, leading to a substantial reduction in both total STAT3 protein levels and phosphorylated protein levels. Consequently, the activity of the IFN-γ-JAK-STAT-IRF1 signaling pathway was suppressed, resulting in a reduction in PD-L1 expression. These discoveries provide valuable insights into the potential use of TPL in tumor immunotherapy.

## Toxicologic mechanisms of TPL

**Fig. 3:**
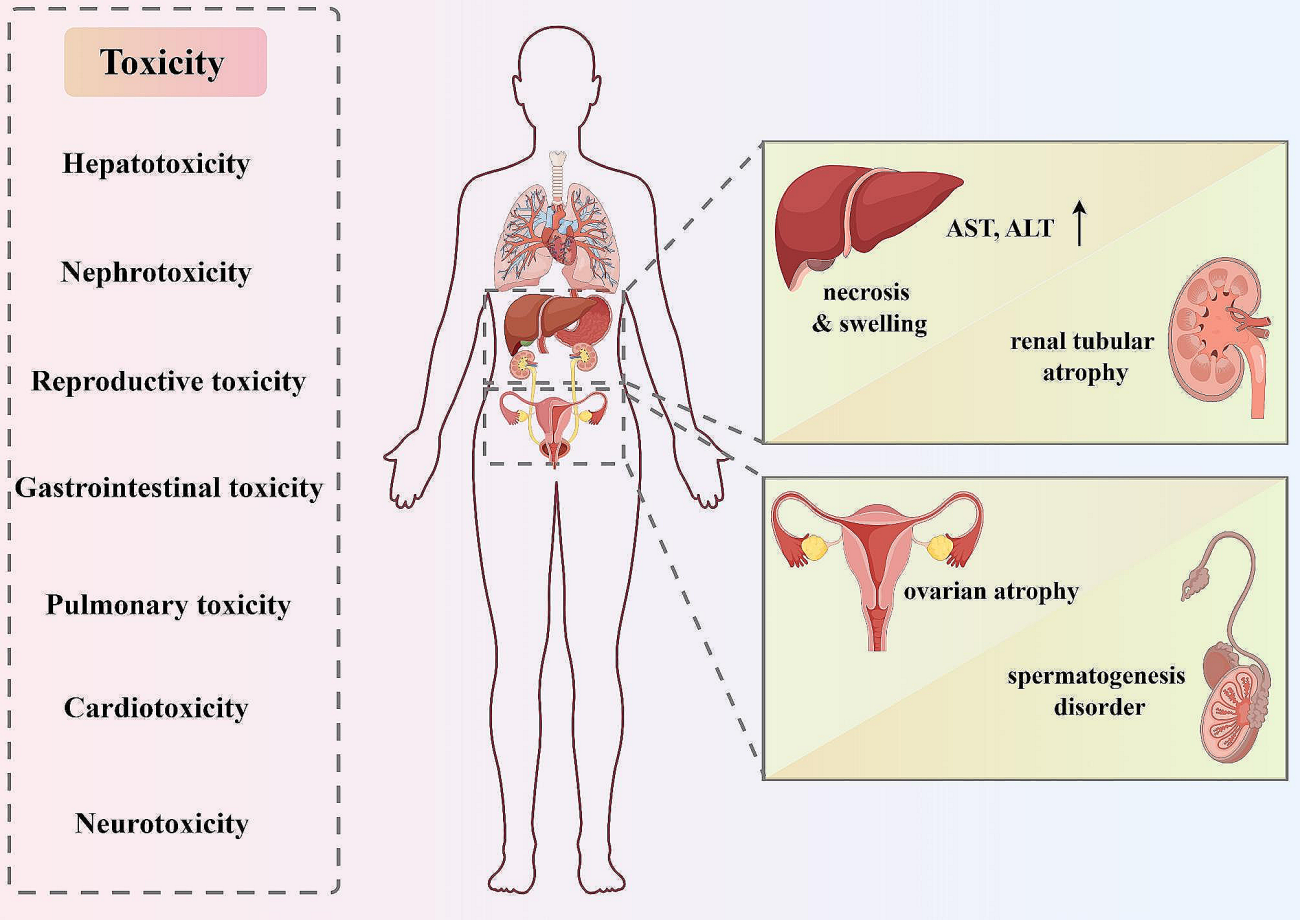
The relevant toxicities associated with TPL. TPL can induce various toxicities, and the figure briefly summarizes the toxicological mechanisms underlying the hepatotoxicity, nephrotoxicity, and reproductive toxicity caused by TPL exposure

TPL exhibits certain toxic side effects (Fig. 3), such as hepatotoxicity, nephrotoxicity, reproductive toxicity, gastrointestinal and pulmonary toxicity, cardiotoxicity, as well as neurotoxicity, which to a certain extent limit its clinical application prospects [112, 139, 140].

Hepatotoxicity and nephrotoxicity represent one of the primary toxic side effects of TPL. Animal studies have demonstrated that TPL can induce liver and kidney tissue injury in mice and rats, manifested by elevated serum levels of aspartate aminotransferase (AST) and alanine aminotransferase (ALT), hepatocellular necrosis and swelling, as well as renal tubular atrophy and other pathological changes [141]. Mechanistic studies indicate that TPL-induced hepatotoxicity and nephrotoxicity may be associated with apoptosis, oxidative stress, and inflammatory responses triggered by TPL [142–145]. Transcriptomic analyses suggest that the PI3K/AKT, MAPK, TNFα, and p53 signaling pathways are crucial steps in TPL-induced hepatocellular apoptosis [146]. Acylcarnitines have been identified as potential early biomarkers for TPL-induced liver injury. The abolishment of hepatic P450s leads to a loss of TPL metabolic capacity in the liver, exacerbating its toxicity. This implies that in clinical applications, P450 inhibition/inactivation would result in severe TPL-related toxic side effects [147]. Studies have shown that TPL-induced hepatotoxicity is associated with the disruption of the PPARα-IL6-STAT3 axis, and the PPARα agonist fenofibrate can reverse this liver injury [148]. Furthermore, TPL may also contribute to liver and kidney damage by aggravating oxidative injury through the Nrf2-mediated antioxidant response [149–151].

Reproductive toxicity is another important toxicological characteristic of TPL. Studies have found that TPL can lead to an increase in macrophages and inflammatory responses within the testes of mice [152]. In spermatogenic cells, particularly Leydig and Sertoli cells of TPL-treated mice, there is an upregulation of reactive oxygen species (ROS) signaling and downregulation of pathways associated with spermatogenesis. The dysregulation of these signaling pathways may represent the underlying mechanism of TPL-induced testicular toxicity [152]. In female rats, TPL treatment can cause ovarian atrophy and impair the developmental potential of oocytes [153, 154]. TPL may also have adverse effects on embryonic development, such as developmental delay and teratogenicity [155]. The toxicological mechanisms of TPL partially overlap with its antitumor mechanisms, such as in certain signaling pathways, oxidative stress, and apoptosis induction. However, there are also notable distinctions in terms of specificity, modulation of pathway activities, and dosage requirements. Delineating these differences is crucial for the clinical application of TPL and mitigating its toxic side effects.

TPL exhibits a wide range of metabolic pathways in vivo, primarily undergoing metabolism in the liver, with partial metabolism occurring in the intestinal tract [156, 157]. TPL is mainly excreted from the body via bile and urine .

Studies have demonstrated that cytochrome P450 enzymes can metabolize TPL into metabolites with reduced activity, and the GSH conjugation pathway also contributes to the detoxification of TPL [158]. This provides a reference for the clinical application of TPL. Mechanistically, CYP3A4 and CYP2C19 may be involved in the hepatic metabolism of TPL in humans, with CYP3A4 being the primary isoenzyme responsible for its hydroxylation [159, 160]. Triptolide was converted to four metabolites (M-1, M-2, M-3, and M-4) in rat liver microsomes and three (M-2, M-3, and M-4) in human liver microsomes.

A study identified 10 metabolites of TPL in rat urine, 4 metabolites in rat liver microsome incubations, and one metabolite in rat gut microbiota incubations using mass spectrometry after TPL administration [161]. Among these different systems, the metabolic reactions of TPL involved hydrolysis, hydroxylation, as well as conjugation with sulfates, glucuronides, and GSH. The structures of these metabolites were characterized as 17-hydroxytriptolide, 16-hydroxytriptolide, tripdiolide, and 15-hydroxytriptolide [162]. In rats, there is a significant gender difference in the quantities of TPL metabolites. The major metabolite detected in the urine, feces, and bile of female rats was the monohydroxylated TPL sulfate conjugate, whereas only trace amounts of the monohydroxylated TPL sulfate conjugate were detected in male rats [163].

## Clinical application of TPL

The toxic side effects of TPL have significantly limited its clinical application. However, numerous strategies to mitigate these adverse effects are currently under investigation. While the majority are still in the preclinical research stage, a small number have advanced to clinical trials, holding promise as potential new antitumor therapeutics.

### Drug combination

Table 3 shows that when combined with other drugs in small doses, TPL demonstrates promising synergistic antitumor effects and enhances the tumor’s sensitivity to the drug. The impact of low levels of TPL on platinum-based anticancer drugs in lung cancer cells has been investigated. Low levels of TPL minimally affected the proliferation of A549 and HTB182 cells but significantly augmented cisplatin-induced inhibition of cell growth [164]. TPL treatment increased the phosphorylation of ataxia telangiectasia mutant (ATM) at Ser^1981^ and elevated DNA double-strand breaks (DSBs) [165]. Concurrently, TPL decreased CHK1 phosphorylation at Ser^317/345^, diminishing the survival rate of cells under DNA damage conditions [166–169]. This mechanism enhances cisplatin-induced apoptosis in lung cancer cells by suppressing nucleotide excision repair (NER), thereby effectively amplifying the effects of platinum-based anticancer drugs. In pancreatic cancer, TPL promotes cancer cell apoptosis by inhibiting the postrepair pathway of oxaliplatin-induced DNA damage, concurrently rendering cancer cells more sensitive to such damage [170]. Enzalutamide is indeed a second-generation androgen receptor (AR) antagonist used to treat metastatic castration-resistant prostate cancer (mCRPC) [171]. It works by blocking the binding of androgens (male hormones) to the AR and thereby inhibits the growth and spread of prostate cancer cells that may have become resistant to other treatments. TPL exerts its inhibitory effects on AR by interfering with several key processes [172]. It inhibits the transcriptional activation of AR through CDK7 and XPB. Additionally, TPL disrupts AR binding to the promoter region of AR target genes and inhibits the recruitment of transcription factor IIH (TFIIH) and RNAPII. These mechanisms collectively contribute to the downregulation of AR-mediated transcription and its associated target genes. In vitro studies demonstrated that low doses of TPL combined with enzalutamide synergistically inhibited the survival of CRPC cells. ATM functions as a protein kinase responsible for detecting DNA double-strand breaks and conveying damage signals through the phosphorylation of histone γH2AX [165, 173]. It recruits DNA repair-related proteins to the damage sites and then initiates the repair. TPL has the capacity to reduce ATM expression, thereby hindering the DNA damage response. Brief exposure to TPL enhances the sensitivity of breast cancer cells to doxorubicin, a chemotherapy drug [174]. E-cadherin, a central protein in the epithelial-mesenchymal transition (EMT) signaling pathway, holds significant relevance in the context of EGFR-targeted molecular therapy for tumors [175]. TPL has the capability to impede the EMT signaling pathway, leading to increased expression of E-cadherin and decreased expression of MMP9, SNAIL, and vimentin. It effectively enhances the sensitivity of the drug-resistant lung adenocarcinoma cell line A549 to gefitinib. When TPL is combined with gefitinib, it shows significantly improved efficacy compared to the drug gefitinib alone [176]. Furthermore, the combination of TPL with erastin, an inhibitor of SLC7A11, heightens the susceptibility of cancer cells to ferroptosis. This synergistic combination exhibits potent antitumor effects both in vitro and in a nude mouse model [109].

**Table 3:** Combination of TPL with other anticancer drugs

Medications	Dose	Pathway of Action	Cancer Types	References
Triptolide+ Cisplatin	Triptolide **10ng/ml**-A549, **5ng/ml**-HTB182 **1ng/ml**-CRL5810, CRL5922 Cisplatin **5μM**	capase3 activation↑ ATM phosphorylation at Ser^1981^↑ CHK1 phosphorylation at Ser^317/345↓^ Nucleotide excision repair↓	Lung Cancer	[164]
Triptolide+ Oxaliplatin	Triptolide **50nM** Oxaliplatin **0–10μM**	DNA damage repair↓	Pancreatic Cancer	[170]
Triptolide+ Enzalutamide	Triptolide **75 μg/kg** Enzalutamide **25 mg/kg**	TFIIH and RNA Pol II recruitment↓	Castration-resistant Prostate Cancer	[171]
Triptolide+ Doxorubicin	Doxorubicin was used after pretreatment with **20/40 nM** of Triptolide for 3 h	ATM↓ DNA damage response↓	Breast Cancer	[174]
Triptolide+ Gefitinib	Triptolide **2ng/ml** Gefitinib **1.25 μg/ml**	Epithelial-Mesenchymal Transition↓	Lung Adenocarcinoma	[176]
Triptolide+ Erastin	Triptolide **0.1 mg/kg** Erastin **10 mg/kg**	NRF2/SLC7A11↓	Head and neck squamous cancer	[109]

Antibody-drug conjugates (ADCs) are monoclonal antibodies that are precisely linked to cytotoxic drugs through specialized linkers. This linkage allows for highly targeted drug delivery, enhancing the precision of treatment. Wei et al. developed three ADCs (L1-TL, L2-TL, and L3-TL) for TPL, which exhibited high efficacy against HER2-targeted tumors both in vitro and in vivo [177]. They employed carbamates as linkers and integrated PEG (polyethylene glycol) and distinct PEG chains to enhance the hydrophilicity of the drug linker. Additionally, they used disulfide rebridging to attach the drug linker to trastuzumab site-specifically. This targeted delivery strategy enhances the drug’s efficacy by accurately targeting tumors while reducing nontargeted side effects.

### Drug delivery

**Fig. 4:**
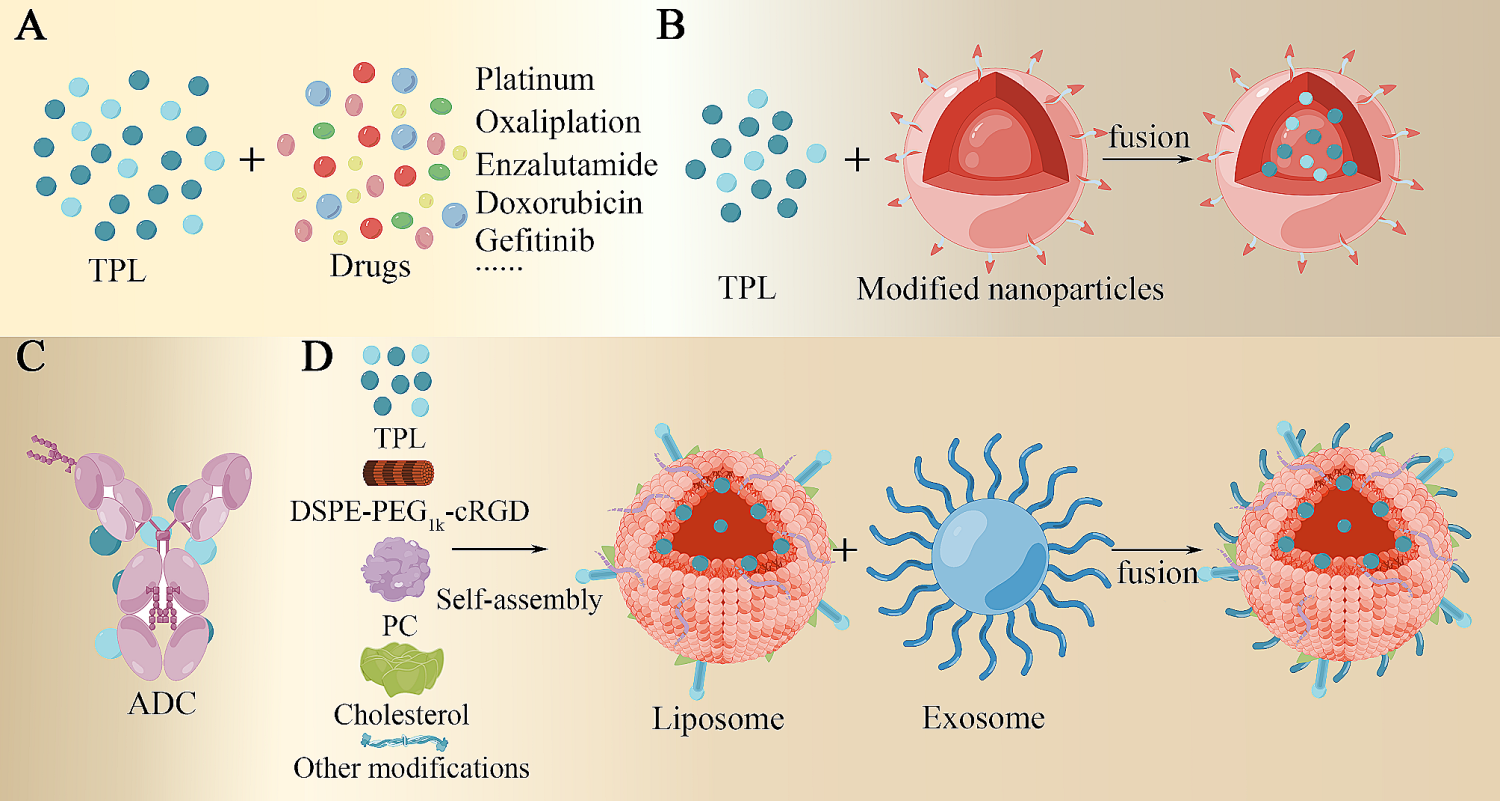
A study of standard forms of application of TPL. (**A**) TPL in combination with other drugs. (**B**) TPL binding to modified nanoparticles. (**C**) TPL-conjugated antibodies enable targeted co-delivery. (**D**) TPL binding to liposomes and exosomes

Researchers have designed a variety of application forms that take full advantage of TPL’s antitumor properties, as shown in Fig. 4. The development of specialized TPL delivery systems or drug delivery methods can also help reduce TPL side effects. For example, a TPL-loaded micellar system, created using a thin-film method for controlled delivery, minimizes drug absorption by the liver and enhances its distribution in the ovary. This approach significantly improves the antitumor effectiveness of TPL in treating ovarian cancer [178]. Exosome-conjugated drugs are another standard mode of delivery. The TPL exosome delivery system (TPL-Exos) possesses exosome-like properties and exhibits a high drug encapsulation rate. Compared to free TPL, TPL-Exos have demonstrated more substantial antitumor effects on ovarian carcinoma SKOV3 cells and an attenuated toxic effect on normal cells [179]. Li et al. fused CD47-expressing tumor exosomes with cyclic arginine-glycine-aspartate acid (cRGD)-modified liposomes, creating a hybrid nanoparticle termed miR497/TPL-HENPs [180]. This innovative delivery system codelivers miR497 and TPL, leading to the effective inhibition of the PI3K/AKT/mTOR pathway, increased ROS production in tumor cells, and the induction of macrophage polarization from the M2 to M1 phenotype. This approach demonstrates a partial overcoming of cisplatin resistance in ovarian cancer. Gu et al. employed exosomes derived from human umbilical cord mesenchymal stromal cells (hUCMSCs) coupled with cRGD to encapsulate TPL [181]. This strategy resulted in the creation of a biomimetic targeted drug delivery system known as cRGD-Exo/TP. Experimental findings indicated that cRGD-Exo/TP exhibited exceptional tumor targeting capabilities and substantially extended the half-life of TPL, all while demonstrating minimal toxicity.

Nanoparticles offer advantages in drug delivery due to their ordered internal structure and large surface area. Researchers have explored various nanoparticle-based strategies to enhance the effectiveness of TPL while reducing its systemic toxicity. One approach is the development of tumor pH-sensitive nanoformulations of TPL coated with folic acid, which specifically target hepatocellular carcinoma (HCC) cells overexpressing the folic acid receptor. These formulations improve TPL efficacy while minimizing systemic toxicity [182]. Innovative nanoparticles of calcium phosphate-conjugated TPL-loaded liposomes, referred to as TP@Lips-Ca/P, have been developed. These nanoparticles demonstrate improved antitumor effects against ovarian cancer cells, specifically SKOV-3 cells, while mitigating reproductive toxicity [183]. Dextran, a highly effective intracellular delivery vector responsive to KRAS, has been linked with TPL to create DEX-TP. This formulation enhances efficacy and quicker cell sedimentation rates in KRAS-mutated cells. DEX-TP selectively delivers TPL to KRAS-mutated cancer cells, thereby reducing cytotoxicity-induced tumor immune exhaustion [184]. Nie et al. developed supramolecular nanoparticles, known as TSCD/MCC NPs, which were loaded with TPL and costimulated under pH 5.0 and acetylcholine esterase (AChE) conditions. This approach led to a cumulative TPL release rate exceeding 90% within 60 h [185]. In cellular experiments, it was observed that this approach demonstrated high toxicity toward four different cell lines, namely A549, SW480, MCF-7, and HL-60 while showing low toxicity toward the normal lung epithelial cell line BEAS-2B. This promising outcome suggests potential clinical applications. An injectable thermo-responsive nanogel encapsulating TPL achieved localized, precise treatment of breast cancer in vitro [40]. This approach exerts a ‘two-strike’ effect. Firstly, it enhances the cytotoxicity of TPL against breast cancer cells. Secondly, TPL suppresses tumor angiogenesis. This methodology effectively augments the antitumor efficacy of TPL.

Furthermore, TPL-loaded γ-cyclodextrin metal-organic framework (TPL@CD-MOF) nanoparticles have been engineered to improve the solubility, bioavailability, and antitumor effects of TPL against hepatocellular carcinoma. TPL@CD-MOF exhibits significantly higher antitumor activity in vitro and in vivo than free TPL [186]. A multifunctional nanoplatform encapsulating TPL with HA modification (HAOPTS) has been developed, effectively targeting tumors, inhibiting the EMT process in tumor cells, and suppressing the development of metastatic tumors [187]. A locally administered formulation of bone-targeted ALE-modified lipid/oil-based TPL nanoparticles has been developed to treat bone metastases. This approach helps reduce the potential for systemic toxicity while still exerting anticancer effects [188]. When combined with paclitaxel or docetaxel, even at relatively low doses, Taxane-Platinum-Nanoparticle (TPN) exhibits potent chemosensitizing effects. Liquid crystalline nanoparticles (LCNs) containing TPL and surface-loaded with small interfering RNAs (siRNAs) targeting TNF-α and IL6 have been employed to achieve a multitargeted approach for modulating the progression of psoriasis [189].

A TPL membrane protein chimeric liposome (TPL@MPLP) has been engineered, resulting in the cessation of cell proliferation, apoptosis, and necrosis through the RIPK1/RIPK3/MLKL signaling pathway. This formulation significantly diminishes the toxic impact of TPL on the liver and kidneys while enhancing its inhibitory effect on hepatocellular carcinoma cells [190]. (SFN + TPL)@CPLCNPs, a bionanoparticle coloaded with the liver cancer chemotherapeutic agents sorafenib (SFN) and TPL, employ platelet (PLT) membranes to facilitate prolonged circulation and tumor targeting. This approach achieves a more effective synergistic effect by lowering the dosage of sorafenib while still maintaining strong anticancer performance [191]. Nanoparticle NTPL-LBA, designed with lactobionic acid (LBA), which selectively targets cancer cells with overexpressed β-D-galactose receptors, releases TPL in its active form within cancer cells. It leads to cancer cell death while minimizing toxicity to noncancerous tissues. This formulation has shown effectiveness in models of pancreatic cancer tumors [192]. P/T-LPNs, which are lipid-polymer hybrid nanoparticles containing both paclitaxel (PTX) and TPL, display synergistic antitumor effects in human lung cancer cells. The most effective weight ratio of PTX to TPL in these nanoparticles was found to be 5:3 [193]. Mesoscale nanoparticles (TP-MNPs) loaded with TPL, designed with renal targeting capability and controlled release properties, have been developed. These nanoparticles have effectively treated renal ischemia-reperfusion injury (IRI) while causing minimal hepatotoxicity, reproductive toxicity, and immunotoxicity [194]. A nanoformulation of sodium hyaluronate-coated TPL exhibits improved absorption by breast cancer cells, enhancing efficacy while reducing systemic toxicity [195]. Conjugating glucose to TPL to create GSH conjugates results in compounds that exhibit increased selectivity and potency in eradicating cancer cells, especially in hypoxic conditions. This approach effectively addresses chemoresistance induced by hypoxia within the tumor microenvironment [196]. Yang et al. developed a tretinoin nanoemulsion gel for transdermal administration, aiming to reduce the toxic effects of TPL [197].

### Clinical trial

The University of Minnesota researchers, led by Banerjee et al., synthesized a water-soluble analogue of TPL known as Minnelide by introducing a phosphate ester group. In vivo, this phosphatase enzyme cleaves this phosphate ester group, resulting in the release of the intermediate O-hydroxymethyl formaldehyde and the original TPL compound. Minnelide has demonstrated effectiveness in diminishing the growth and metastasis of pancreatic tumors [198]. Currently, it is undergoing phase II clinical trials for the treatment of advanced pancreatic cancer [12]. Recent studies have revealed noteworthy antitumor efficacy of Minnelide in malignancies, such as pancreatic cancer, liver cancer, CRPC, and ovarian cancer [198–201]. Combining Minnelide with conventional chemotherapeutic drugs allows for dose reduction while maintaining increased efficacy [202]. The prospects of Minnelide’s application are up-and-coming.

Another TPL analog, (5R)-5-hydroxytriptolide (LLDT-8), is a low-toxicity immunosuppressant currently in phase I clinical trials [203]. LLDT-8 has been found to reduce imiquimod (IMQ)-induced psoriasis-like dermatitis by downregulating IL-36α expression and blocking the IL-36α pathway [204]. Treatment with LLDT-8 leads to reduced Toll-like receptor 4 (TLR4) expression in bone marrow-derived dendritic cells (BMDCs). It also results in the phosphorylation of IκBα and the subsequent nuclear translocation of NF-κB [205]. LLDT-8 alleviates LPS-induced acute lung injury (ALI). Additionally, Wang et al. demonstrated that LLDT-8 effectively overcomes multidrug resistance mediated by P-glycoprotein and exhibits robust antitumor activity, especially in cases of drug resistance mediated by P-glycoprotein in various cancer types [206].

## Conclusions

The growing worldwide prevalence of cancer has underscored the pressing requirement for precise and efficacious therapies in recent years. Regrettably, numerous malignant tumor cells have developed resistance to current treatments, compounding the complexity of this issue. Nevertheless, TPL has emerged as a promising contender, exhibiting established antitumor effects in diverse cancer cell types. As researchers delve further into the mechanisms underlying TPL’s actions, its potential in antitumor therapy becomes progressively apparent.

TPL exerts its anticancer effects through intricate signaling pathways, including the NF-κB, STAT3, TRAIL, and Wnt/β-catenin pathways. It accomplishes this by binding to RNA polymerase, effectively inhibiting transcription, resulting in substantial epigenetic modifications within tumor cells and cell death. Furthermore, TPL has been demonstrated to trigger various forms of cell death, including apoptosis, autophagy, pyroptosis, and ferroptosis. These multiple mechanisms offer diverse potential avenues for therapeutic intervention.

While TPL has shown remarkable potential in antitumor applications, its considerable toxicity and limited water solubility have presented challenges in clinical use. Researchers have investigated various strategies to address these issues, including encapsulation techniques and combination therapies, to alleviate TPL toxicity while maintaining its effectiveness. These approaches enhance TPL’s solubility, bioavailability, and targeted delivery, ultimately improving its therapeutic profile and minimizing adverse effects. An in-depth analysis of the toxicological mechanisms and metabolic pathways of TPL may provide effective data support for its clinical applications.

While experimental studies have provided insights into the influence of TPL on downstream signaling pathways and associated factors, there is still a need for a more profound understanding of how TPL sensitizes these factors, consequently enhancing their therapeutic effectiveness. Further exploration of the mechanisms behind TPL-induced sensitization could yield valuable insights and potentially open the door to innovative combination therapies that leverage the distinctive properties of TPL.

The clinical use of TPL presents significant potential in antitumor therapy. Its multifaceted mechanisms of action, coupled with ongoing endeavours to address its constraints, have the potential to reshape cancer treatment paradigms. By harnessing the anticancer capabilities of TPL and integrating them with other therapeutic approaches, healthcare professionals and researchers can explore novel pathways for personalized and targeted treatments that combat drug resistance and enhance patient outcomes.

In conclusion, TPL is a valuable asset in the battle against cancer, boasting diverse antitumor mechanisms and the potential for targeted delivery systems. Ongoing research into the therapeutic uses of TPL, including its combination with existing treatments, provides optimism for more effective and precise cancer therapies. As our comprehension of TPL’s mechanisms of action continues to evolve and novel delivery methods are developed, the groundwork is laid for its eventual clinical translation, opening new horizons in antitumor research.

## Data Availability

No datasets were generated or analysed during the current study.
